# The Impact of Knee and Ankle Injuries on National Basketball Association Player Performance Post-injury

**DOI:** 10.7759/cureus.58943

**Published:** 2024-04-24

**Authors:** Justin Ceasar, Andrew Yeich, Peter Shafeek, Kushagra Kumar, Robert P Olympia

**Affiliations:** 1 Physical Medicine and Rehabilitation, Penn State College of Medicine, Hershey, USA; 2 Medicine, Penn State College of Medicine, Hershey, USA; 3 Emergency Medicine, Penn State College of Medicine, Hershey, USA; 4 Emergency Medicine and Pediatrics, Penn State Health Milton S. Hershey Medical Center, Hershey, USA

**Keywords:** sports injury, nba, knee injury, game performance, basketball, ankle injury

## Abstract

Introduction: Athletes in the National Basketball Association (NBA) are subjected to high levels of mechanical stress increasing their risk of injury. The purpose of this study was to see how certain lower extremity injuries affect in-game performance in relation to each NBA athlete's demographics. The hypothesis was that NBA players' post-injury performance would differ depending on their demographics and the type of injury sustained.

Methods: Descriptive epidemiology study of NBA injury list designations from the 2010/2011 season to the 2018/2019 season. About 255 lower leg injuries that met the inclusion criteria were selected from the injury lists spanning from the 2010/2011 season to the 2018/2019 season. These included ligamentous knee injuries, knee sprains, knee strains, knee hyperextensions, patellar injuries, ankle injuries, and Achilles injuries. The change in performance was determined by comparing mean game scores before and after injury with single-tailed, heteroscedastic t-testing and 95% confidence intervals for mean values.

Results: An overall statistically significant decrease in mean game score from 9.82 to 8.75 was seen in all included players (p = 0.01). Only athletes taller than the mean height (199.85 cm; p = 0.01) and heavier than the mean weight (101.63 kg; p = 0.02) showed a significant decline in performance. Ankle and knee injuries both resulted in a significant loss in game score (p = 0.04), with ankle injuries resulting in a greater average decline (-1.76 post-injury) than knee injuries (-1.34 post-injury).

Conclusions: These findings suggest that treatment regimens should reflect the type of injury and demographics of the specific NBA player injured. Further research is warranted to determine if treatment may be more efficacious when streamlined based on player size and injury type.

## Introduction

Athletes in the National Basketball Association (NBA) are at an increased risk of injury due to the high-speed nature and physical demand of the sport in combination with the frequency of minutes played in back-to-back games. Excluding additional playoff games, these players are typically subjected to 82 games over the course of six months, averaging close to three games per week [1]. For this reason, NBA players often sustain a variety of injuries that can cause them to sit out of basketball activities for extended periods and, sometimes, even end an athlete’s career [2-5]. Common injuries in the NBA include stress fractures, ligament tears, hamstring strains, and lumbar strains, with lower extremity injuries accounting for 62.4% of all injuries [6,7]. Furthermore, lateral ankle sprains alone have been shown to account for roughly 13.2% of orthopedic injuries within the NBA [6].

Data pertaining to other professional sports suggests that such injuries can weigh greatly on an athlete’s performance as well as their team’s performance [8]. For example, a 2013 study on injuries in European football displayed a significant relationship between increased injuries and team performance [9]. The study demonstrated that those teams with more injuries to their players in a single season tended to display worsened team performances and league position finishes. In addition, a 2017 study concluded that athletes who are inclined to play more minutes and more games expose themselves to an accumulated workload that is placed on their extremities, leading to an increased risk of injury [10]. 

Previous research has examined the impact of sustaining a specific injury and its relation to a player’s performance utilizing tools such as player efficiency rating (PER) [2-5,7,11-13]. In particular, a 2021 study showed a decline in in-game performance following Achilles tendon ruptures among Women’s NBA athletes [14]. Other studies have focused on identifying risk factors that could predispose athletes to sustaining injuries, which included sleep deprivation and busy game schedules [10,12]. Further research has even examined the effects of specific injuries in other non-basketball professional sports leagues [5,15,16]. Despite this focus on what could cause a player to sustain an injury, there appears to be a gap in research relating to the impact that lower extremity injuries could have on an individual NBA player’s performance.

To our knowledge, very few published studies have collectively examined the effects that various lower extremity injuries sustained by NBA players may have on their post-injury in-game performance. With lower extremity injuries making up a majority of NBA injuries, we believe that investigating such a topic could help predict if certain injuries may allow a player to return to their previous levels of play [6,7]. The aim of this study, therefore, was twofold (1) to investigate the relationship between common knee, ankle, and Achilles injuries with player performance, and (2) to determine the potential need for specific rehabilitation plans that better suit individual players based upon their demographics, such age, size, and position played. We hypothesized that players who return to play after recovering from a lower extremity injury would show an overall decrease in performance compared to pre-injury performance. Of note, this article was previously presented as a meeting poster at the American College of Sports Medicine 2023 Annual Meeting and World Congress on May 31, 2023.

## Materials and methods

Beginning with a list of every injured list (IL) activation in the NBA from the 2010-2011 season to the 2018-2019 season, injuries were carefully selected by four reviewers to mitigate confounding variables and identify those that met inclusion criteria. Inclusion criteria consisted of (1) all ligamentous and meniscal knee injuries; (2) strains, sprains, and hyperextensions of the knee; and (3) patellar, ankle, and Achilles injuries in the NBA between October 3, 2010 and September 28, 2019. Exclusion criteria consisted of injuries before or after which the player had not played 20 games (“insufficient data”), any non-ankle and non-knee related injuries (i.e., calf strains or foot injuries), injuries with other concomitant injuries occurring during either 20-game period (“recent concomitant injuries”), and injuries of players traded to another team during either 20-game data collection period (“traded”). IL activations occurring during the 2019-2020 season to the present day were excluded to avoid any confounded data related to COVID-19 infections, exposures, and quarantines. The list was obtained from a professional database that tracks and records all IL movements throughout each NBA season [17].

**Figure 1:**
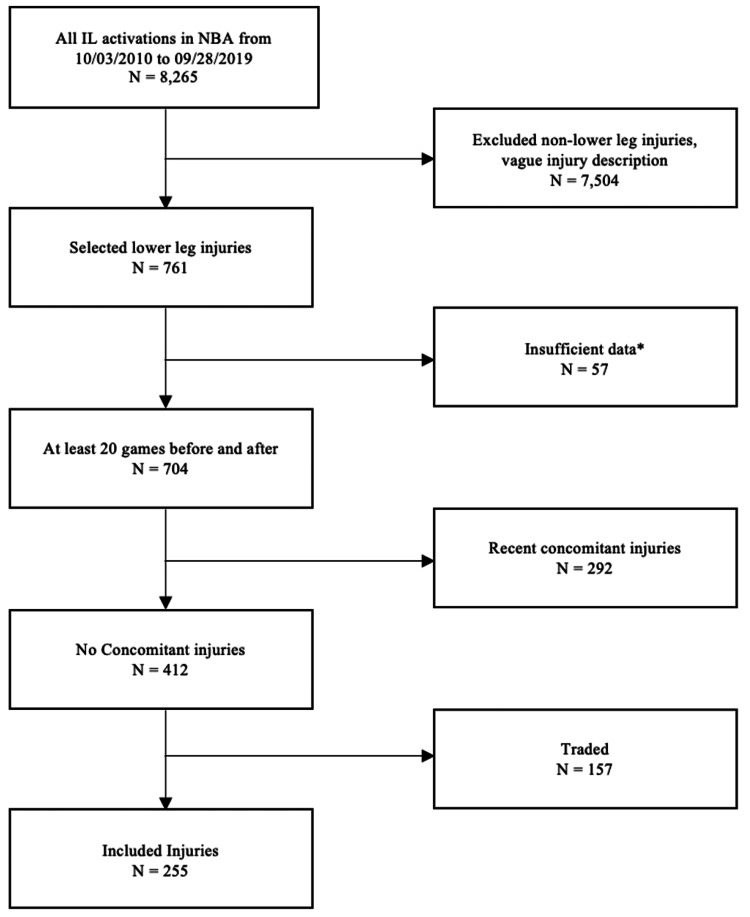
Flowchart of Injury Selection Process Inclusion criteria were (1) all ligamentous and meniscal knee injuries; (2) strains sprains, and hyperextensions of the knee; and (3) patellar, ankle, and Achilles injuries in the NBA between October 3, 2010, and September 28, 2019. Exclusion criteria were injuries before or after which the player had not played 20 games (“insufficient data”)*, injuries with concomitant injuries occurring during either a 20-game period (“recent concomitant injuries”), and injuries of players traded to another team during either data collection period (“traded”).

The process seen in Figure 1outlines the manual selection process along with the number of players meeting each criterion. Only isolated injuries involving the knee and ankle were considered for selection. Additionally, those without an exact labeled diagnosis, such as “sore left knee,” were excluded to allow for stratification by injury type. Players who were injured in their rookie year before playing 20 games were excluded due to insufficient pre-injury data. To prevent the data from reflecting the effects of multiple injuries, players who were placed on the IL a second time in the 20 games preceding or following their selected injury were removed. As a team’s overall performance can impact the statistical performance of an individual player, injuries in which the player was traded to a different team at any point during the pre- or post-injury 20-game data “collection period” were also excluded.

Every injury meeting the criteria was then manually located in the individual players’ statistical record within a professional sports analysis and statistics website [18]. From here, the demographic information of each player was recorded, and the average game scores during the 20 games preceding the injury and the 20 games following were calculated. Demographic information collected included each player’s age, team, position, previous years of NBA experience, height, and weight all estimated from the time of the injury. Data was collected into both Google Sheets and Microsoft Excel for further analysis. Players who did not start or sub into game(s) during the data collection period, but were not injured, had their collection period extended to encompass the immediate 20 games in which they featured. Game score is an aggregate statistic widely used in NBA analysis that is calculated by game score = points scored + (field goals × 0.4) + (field goals attempted × (-0.7)) + ((free throws attempted - free throws made) × (-0.4)) + (offensive rebounds × 0.07) + (defensive rebounds × 0.3) + steals + (assists × 0.7) + (blocks × 0.7) + (personal fouls × (-0.4)) - turnovers [18-21]. The game score was chosen as the unit of measurement in this study given it encompasses most major statistical categories that highlight the performances of NBA athletes. All injury data was cross-referenced for accuracy by three additional reviewers before progressing through each stage of data curation. Players who played more than one position in a season were assigned the position at which they played the most games during the season of focus. Data was then stratified by player age, weight, height, position, injury type, and whether the data collection periods occurred during the same season or overlapped multiple seasons. To evaluate the null hypothesis for each individual data subcategory, the mean game scores before and after injury were compared using single-tailed, heteroscedastic t-testing. To evaluate the magnitude of game score change between data subcategories, the mean percent change in game score was calculated followed by 95% confidence intervals for the mean value. Inter-subcategory t-tests were then performed following the same parameters. This study was exempt from Institutional Review Board (IRB) submission as per Pennsylvania State University’s IRB guidelines.

## Results

The demographics and process of selection have been outlined in Figure 1. Of note, 21 players were excluded due to retirement after injury. In looking at player statistics for those with leg injuries from October 2010 to September 2019, 255 players were included in the study. Of these 255 players, 148 sustained leg injuries with which their game performance could be analyzed based on pre- and post-injury within the same normal 82-game season plus possible playoff participation. Table 1** **displays the demographic breakdown of the injuries that met the inclusion criteria. The subgroup of injuries spanning only one season was highly comparable to those spanning multiple seasons. When stratified by age, 115 of the players studied were below 26 and 140 were above (mean age <26 = 22.87 and ≥26 = 29.98; Table 1).

**Table 1:** Demographic Information for Injured Players by Subcategories The leftmost column considers all included injuries, and the rows to the right are divided into subgroups based on player characteristics at the time of injury. “Same season only” contains only injuries in which the player completed the 20-game collection periods during the same season, while “Different season only” contains only injuries in which the player did not. “PG” contains only players playing point guard as their primary position during the season that the injury took place while “SG” refers to shooting guards, “SF” to small forwards, “PF” to power forwards, and “C” to centers. The rows refer to mean player characteristics at the time of injury. *Refers to significant differences between these two categories after calculating the 95% confidence interval.

	All injuries	Same season only	Different season only	Age < 26	Age ≥ 26	Weight < 100 kg	Weight ≥ 100 kg	Height < 200 cm	Height ≥ 200 cm	PG	SG	SF	PF	C	Knee	Ankle
N	255	148	107	115	140	126	129	106	149	58	46	46	53	54	77	165
Mean age	26.22	26.19	26.31	22.87	28.98	26.12	26.33	25.95	26.40	25.95	26.16	26.59	25.81	26.67	25.82	26.38
Mean years in the NBA	5.35	5.28	5.44	2.55	7.65	5.06	5.63	4.85	5.70	4.57	4.93	5.35	4.88	27.69	5.08	5.41
Mean weight (kg)	101.63	102.18	100.91	102.02	101.33	91.31	111.91	91.12	109.14	86.55	95.08	101.70	109.36	115.70	200.41	201.02
Mean height (cm)	199.85	199.34	200.43	200.91	198.98	193.7	205.94	191.39	207.12	188.31	195.76	202.26	207.14	206.35	101.79	102.07
Mean days injured	72.04	53.74	128.11	76.23	68.61	79.62	64.52	88.23	61.53	83.47	86.70	74.65	68.77	48.81	94.13*	50.35*

**Figure 2:**
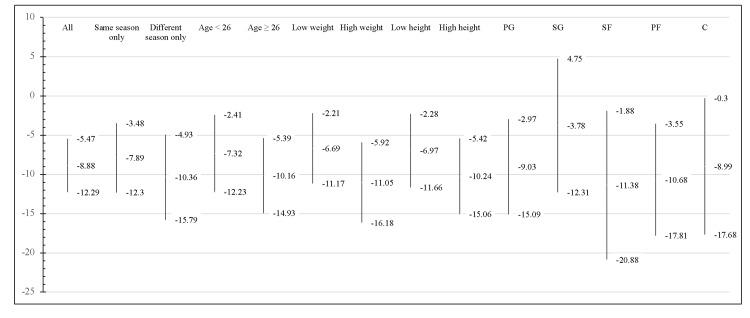
Mean Percent Change in Game Score by Subgroup Percent change in game score following injury was calculated for each injury and averaged to compare between subgroups. The middle value in the range refers to the mean percent change in game score, and the higher and lower values correspond to the upper and lower range of the distribution’s 95% confidence interval. All inter-subgroup comparison p-values are >0.10 (not shown in the figure). Note that “low weight” refers to those players below the mean weight of all players included in the study (101.63 kg) and “high weight” refers to those players above this weight. Likewise, “low height” refers to those players below the mean height of all players included in the study (199.85 cm), whereas “high height” refers to the players above this height. PG: point guards; SG: shooting guards; SF: small forward; PF: power forwards; C: centers

The included injuries were also separated along the 50th percentile of height and weight; the taller and heavier players had similar ages as their shorter and/or lighter counterparts but experienced shorter injury durations. As seen in Table 1, analysis by position yielded variation in height, weight, and injury duration, with point guards (PG) and shooting guards (SG) taking the longest to recover. Players who are guards also tended to be shorter and lighter, on average, than forwards and centers (Figure 2). We found that among these players, centers spent the least overall number of days injured as compared to other position groups. Finally, in utilizing a 95% confidence interval, a significantly lower mean for days injured is seen in players who suffered from an ankle injury (50.35 days) when compared to those who suffered from a knee injury (94.13 days). The other comparisons analyzed did not display statistical significance.

The table below reports the mean game scores for each subgroup before and after injury (Table 2). Collectively the included players saw a statistically significant game score decrease of 1.07 (p = 0.005). Significant declines in post-injury game score averages were also seen among both players returning the same season (p = 0.05) and those whose injuries spanned over multiple seasons (p = 0.02). Both the younger and older age categories demonstrated significant variation post-injury (p = 0.04) and (p = 0.03), but only the heavier and taller players followed suit (p = 0.02) and (p = 0.01). Every position subgroup failed to reject the null hypothesis. Dividing by injury type yielded the same significant p-value of 0.04 for each subgroup, but ankle injuries caused a larger average decrease in game score (decreased from 11.09 to 9.33) than those of the knee (decreased from 9.82 to 8.48).

**Table 2:** Mean Game Scores Before and After Injury The top row considers all included injuries. The rows below are divided into subgroups based on player characteristics at the time of injury using the same descriptors as Table 1. Mean game scores for 20-game periods before and after injury were calculated for each group and compared to the null hypothesis of no significant change in game score using single-tailed, heteroscedastic t-testing to report p-values in the rightmost columns. Note that 101.63 kg was used as the cutoff for weight as this was the overall mean weight of all players included in the study. Likewise, 199.85 cm was used as the cutoff for height as this was the mean height of all players included in the study. PG: point guards; SG: shooting guards; SF: small forward; PF: power forwards

		Mean before injury	Mean after injury	p
Season	All	9.82	8.75	0.01
	Same only	9.82	8.91	0.05
	Different only	9.84	8.55	0.02
Age	<26	9.53	8.54	0.04
	≥26	10.06	8.92	0.03
Weight (kg)	<101.63	10.19	9.26	0.06
	≥101.63	9.45	8.24	0.02
Height (cm)	<199.85	10.34	9.45	0.09
	≥199.85	9.44	8.26	0.01
Position	PG	11.12	9.92	0.10
	SG	9.78	9.23	0.29
	SF	9.19	8.08	0.12
	PF	9.98	8.71	0.08
	C	8.8	7.7	0.08
Injury type	Knee	9.82	8.48	0.04
	Ankle	11.09	9.33	0.04

The chart below plots the mean percent change in game score in a comparison by subgroup (Figure 2). As shown by the wide confidence interval ranges, there was widespread variance in the data even after thorough efforts to mitigate concomitant variables were undertaken, as seen above (Figure 1). For binary categories, t-testing was attempted but failed to identify a significant difference in the magnitude of the percent change in game score between any of the comparisons. Players whose injuries spanned multiple seasons had, on average, a larger percent change (-10.36%) than those who returned the same season (-7.89%). Older, heavier, and taller players also experienced larger proportional declines in performance on average post-injury. Consideration by position noted a much smaller percent change for SG (-3.78%) than the other positions, the next lowest of which being centers (C) (-8.99%). However, the n of each group was low due to exclusion criteria, so this may not accurately represent the difference in performance by position (Table 2).

## Discussion

The analysis of the impact of specific lower extremity injuries on the game scores and in-game performance of 255 NBA players analysis revealed a statistically significant overall decrease in game performance following injury (p = 0.005). With injuries having such crucial, course-altering impacts on a player and their team’s season, having insight into the impact of injuries on player performance could allow teams to better plan for training and rehabilitation. As previously mentioned, lower extremity issues have been shown to represent a majority of NBA injuries, with lateral ankle sprains specifically making up the largest percentage of injuries [6,22]. Therefore, these significant results provide valuable evidence of overall decreased player performance following a large portion of NBA injury types. This provides another layer of evidence for NBA players, teams, and fans to consider when establishing expectations regarding a player’s post-injury performance.

With more time to return to full strength post-injury, some psychological strain may be alleviated on players coming back from time out. Previous research dating back to 1990 demonstrated that athletes with more serious, long-term injuries show significantly more tension, anger, and depressive-like symptoms that may impact their ability to return to previous levels of performance for at least one month after returning from injury [23]. This suggests that players with longer-term injuries may benefit from additional mental health services during their rehabilitation to aid in their return to play. Future research to examine the impact that psychological services may have on the post-injury game scores of NBA players who suffer from various lower extremity injuries may help determine if improvement in this area of treatment is necessary. Moreover, the NBA implemented a new resource in 2018 labeled “a new Mental Health and Wellness program” to aid its players in tackling mental health obstacles that they may face [24]. Ensuring that this resource is available and utilized for NBA players who suffer from lower extremity injuries could benefit their post-injury in-game performance long term. With our data collection period ending after the 2018-2019 season due to confounding COVID-19 infections in more recent data, many of the newer mental health services may not have been fully available to the injured players included in the data pool. It would be beneficial to examine if there was any apparent potential impact of these newer psychological services on a player’s ability to return to pre-injury performance in more recent, post-pandemic seasons.

It is also important to note that significant decreases in game performances were seen in players weighing heavier than 101.65 kg (p = 0.02) and/or taller than 200.58 cm (p = 0.01) but not for players who measured less than 101.65 kg (p = 0.06) and/or shorter than 200.58 cm (p = 0.09), respectively. Previous research has associated an increase in either height or weight with increased injury risk among adolescent athletes. In other words, taller and/or heavier student-athletes have shown higher injury rates [25,26]. Another study demonstrated an increased risk of contact injury in heavier rugby athletes [27]. However, to our knowledge, this is one of the first studies to specifically examine the impact that height and weight have on an NBA player’s ability to return to previous levels of in-game performance following injury. With NBA professionals being some of the tallest and largest athletes in the world, understanding the impacts of size on a player’s ability to return to full playing capacity post-injury could impact the way that NBA medical teams approach their patients. This provides implications for future investigation into different rehabilitation techniques that could be used for heavier or taller players. For example, it may be that larger players may require more rest before returning from injury due to increased strain placed upon their bodies.

One area of focus that did not show a significant decrease in game performance post-injury was between different position groups (Figure 2). Although each individual position group did show a general decrease in performance post-injury, no group was shown to be significant against the null hypothesis (Table 2). Furthermore, there was no significant difference in the statistical decrease between different position groups. This suggests that post-injury treatment plans may be better if streamlined based on other factors such as player size and injury instead of their specific position group. This finding is especially understandable in today’s NBA, as many players play across different positions regardless of their size [28]. Additionally, previous research has indicated that scheduling does play a role in the rate at which players are injured, most notably when back-to-back games are played [10]. This is another factor that may need to be considered when NBA medical teams assess a player’s readiness to return to live gameplay.

Much like organizing players by their position groups, stratifying NBA athletes by age did not produce a significant difference in decreased statistics. Although both players aged under 26 years (p = 0.04) and those aged 26 or older (p = 0.03) did show significant decreases in performance, an insignificant difference exists insignificant difference when directly comparing the two groups of players. This further stresses the potential need to assess each injured professional individually (Figure 2).

In addition to the age difference, players separated by type of injury (ankle vs knee) showed no significant difference in decreased game performance (Figure 2). Both knee and ankle categories displayed the same level of significance in decreased game performance (p = 0.04). Further breakdown of injury type was made difficult due to low injury levels. With a clear significant decrease following each general category of injury represented in this study, it provides an important future direction of investigation. Additionally, further work should also be done to determine if players with multiple injuries throughout their careers suffered from greater drop-offs in their in-game performance levels than those who did not.

It is important to note that a few key limitations do exist pertaining to the research of focus. As this study was a retrospective analysis of past NBA injuries, it may be prone to missing data that could not be accounted for by our researchers. The public database used for data collection may introduce reporting bias; however, it is important to note that this technique has been shown to be reliable in other studies [14,29,30]. The method of study did not allow for the research team to account for other unreported concomitant injuries and external factors such as illness, family factors, and social factors that could affect a player’s day-to-day game performance.

Similarly, injuries that players may have suffered earlier in their careers, such as during their college basketball careers, were unable to be accounted for within the scope of this study. However, to account for as many biases and concomitant variables as possible, many injuries were excluded from the study (Figure 1). Because of these exclusions, the data at hand may not be an entirely representative depiction of NBA injury-related statistics. Additionally, there may be a further impact of injury considering 21 players were unable to be accounted for due to retirement following their respective lower extremity injuries. Future research could utilize a lighter inclusion process, as a larger dataset may allow for a more thorough statistical analysis given this population’s high variation. Finally, the height and weight of each player were recorded at the beginning of the season of their injury, so it is possible that small fluctuations in their actual measurements took place between the time of measurement and their injury. 

## Conclusions

evidence of a statistically significant decrease in game performance following a variety of lower extremity injuries. Additionally, a significant decrease in a player’s post-injury statistics for heavier and taller players suggests that a player’s size may impact their ability to return from injury at full strength. Although limitations exist through exclusion criteria used to eliminate possible confounding variables, these findings provide important directions for future research. Future implications include, but are not limited to, developing more realistic and beneficial treatment plans for injured players, as well as investigating the impact that recurring injuries or the availability of mental health services have on an NBA player’s post-injury in-game performances.
